# An 18‐Year‐Old Congenitally Deaf and Mute Male With Prolonged Constipation and Foreign Body Ingestion Managed Conservatively: A Rare Case Report

**DOI:** 10.1002/ccr3.70894

**Published:** 2025-09-18

**Authors:** Muhammad Hassaan Javaid, Muddassir Khalid, Mohammad Yassin Al Aboud

**Affiliations:** ^1^ Shifa College of Medicine Islamabad Pakistan; ^2^ Nishtar Medical University Multan Pakistan; ^3^ Faculty of Medicine Latakia University Latakia Syria

**Keywords:** case report, conservative management, constipation, deaf–mute, foreign body ingestion

## Abstract

Foreign body ingestion in adults is rare and typically linked to psychiatric or cognitive disorders. We present a rare and challenging case of an 18‐year‐old congenitally deaf and mute male who presented with prolonged constipation and recurrent vomiting. Diagnostic imaging revealed extensive fecal impaction and multiple ingested foreign bodies, which were coins and seeds, and there was no sign of any perforation. Due to the patient's communication barriers, diagnosis and management were delayed and required extra care. The patient was successfully managed with conservative procedures, including manual evacuation under sedation and supportive care, avoiding the need for surgical or endoscopic intervention. This case highlights the importance of a multidisciplinary approach during the management of gastrointestinal conditions in patients with communication disabilities and emphasizes the need for improved healthcare accessibility and communication strategies for the deaf and mute population.

**Summary**

- This case highlights the diagnostic and therapeutic challenges of managing foreign body ingestion in a congenitally deaf and mute adolescent. Communication barriers delayed diagnosis and treatment of severe fecal impaction caused by ingested coins and seeds. A conservative, multidisciplinary approach led to a successful outcome without surgical intervention. It underscores the importance of accessible communication strategies and clinical awareness about care for patients with disabilities.

## 1 Introduction

Ingestion or insertion of foreign bodies into the gastrointestinal tract (GIT) presents a serious clinical issue in the Emergency Department, giving rise to a significant financial burden, morbidity, and mortality [1]. Foreign body ingestion causes almost 1500–1600 deaths in the United States each year [2]. While the issue affects people of all ages, foreign body ingestion is more prevalent in children and uncommon in adults, but typically happens by mistake or in people who have intellectual disability, behavioral difficulties, emotional disturbances, psychiatric issues, or alcohol‐related impaired judgment [3].

The majority of these are known to flow through the GIT without any problems. However, between 1% and 14% need operational removal, and 10%–20% must be removed endoscopically, as suggested as potential intervention techniques in the 2002 American Society for Gastrointestinal Endoscopy (ASGE) guidelines [4]. Constipation is frequently caused by consuming foreign bodies, 11%–20% of such cases each year [5, 6]. A unique and noteworthy instance of an 18‐year‐old male, congenitally deaf and mute, who experienced prolonged constipation and ingestion of foreign bodies was treated conservatively. Due to the patient's linguistic and hearing disability, which acted as a communication barrier, this case is unique and intriguing. This obstacle posed a significant difficulty in both diagnosing and treating the illness. Because it demonstrates how to properly identify and treat the problem in people with disabilities, this article is a valuable addition to the body of literature.

## 2 Case Presentation

An 18‐year‐old male from a low socioeconomic background, congenitally deaf and mute, presented with a 3‐week history of relative constipation and recurrent vomiting. The patient had no prior surgical history or hospitalization. There were no relevant past interventions with outcomes.

### 2.1 Clinical Findings

Physical examination revealed a distended but soft abdomen without signs of peritonitis. Digital rectal examination (DRE) identified a rectum filled with soft stools. There were no signs of peritonitis. The DRE is an important examination, and it rules out any possibility of a rectal mass or other causes of mechanical obstruction (such as anal stenosis, rectal prolapse, rectal intussusception, or rectocele) during diagnosis.

### 2.2 Diagnostic Assessment

**FIGURE 1:**
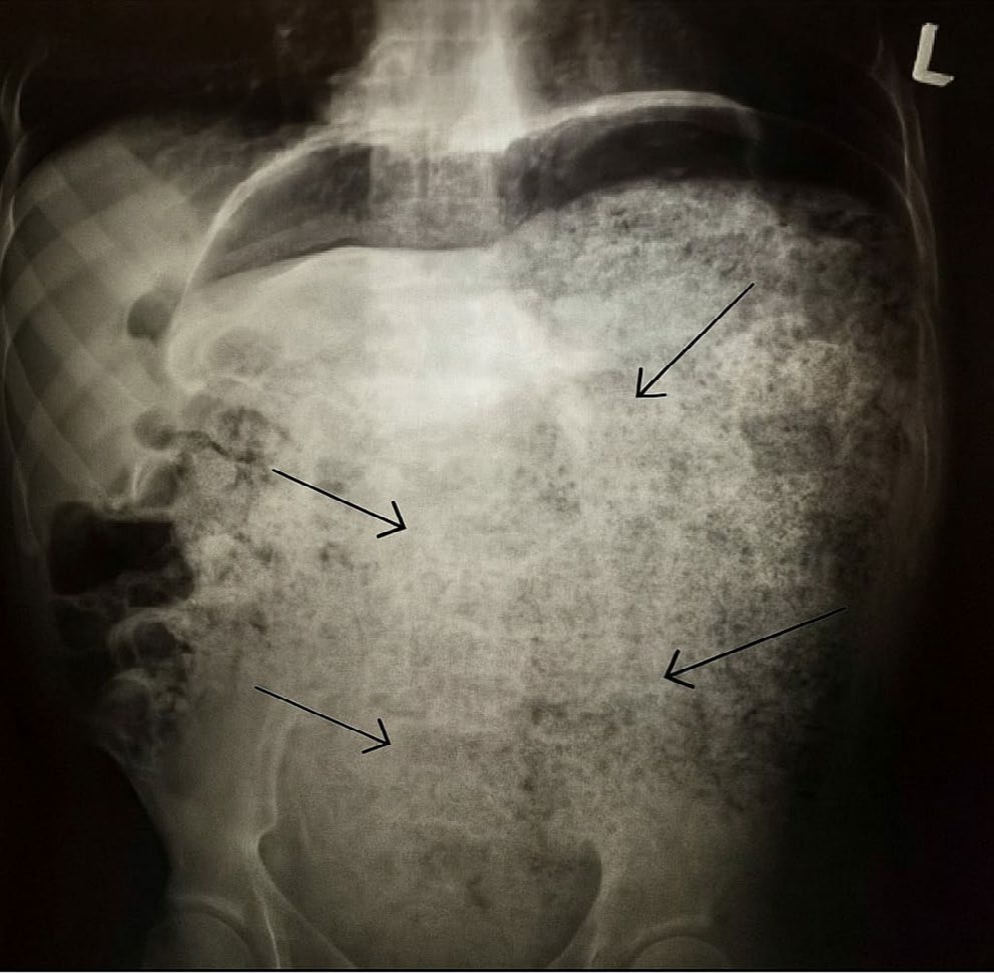
Abdominal X‐ray showing radiopaque foreign bodies (coins) and fecal impaction.

**FIGURE 2:**
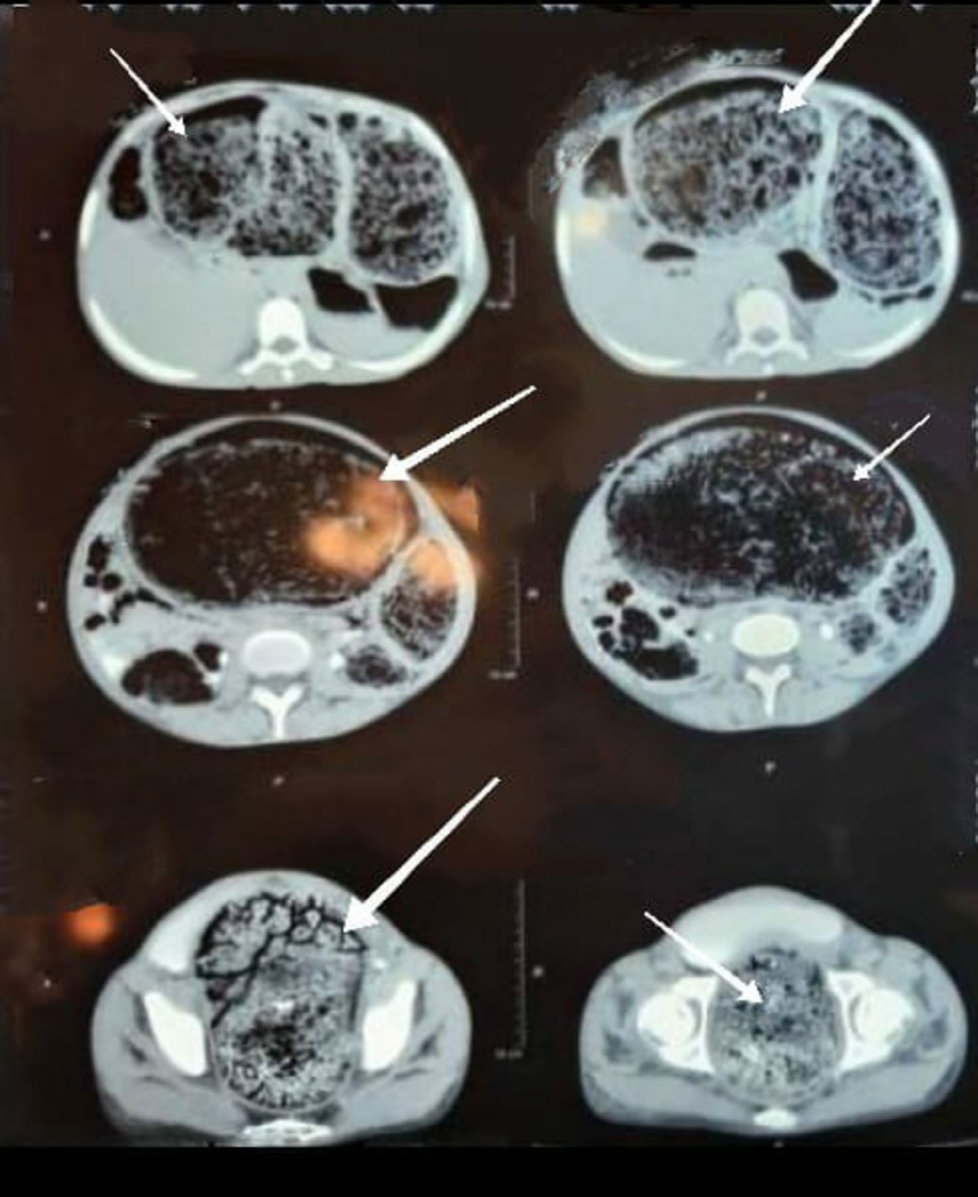
CT scan axial view illustrating colonic dilatation and seed‐shaped foreign material.

Plain abdominal X‐ray and CT scan (Figures 1 and 2) demonstrated colonic dilatation with multiple radiopaque foreign bodies (coins and seeds) and a significant fecal load exceeding 4 kg. No evidence of bowel perforation or ischemia was observed.

Dilatation of the colon, along with foreign body ingestion and fecal impaction, was the diagnosis of the patient. The challenges during diagnostic assessment were due to communication barriers, as the patient was congenitally deaf and mute. The prognosis was that the patient would show gradual improvement with conservative management, along with resolution of abdominal distension and restoration of normal bowel movements, and conservative excavation of foreign bodies.

### 2.3 Management

**FIGURE 3:**
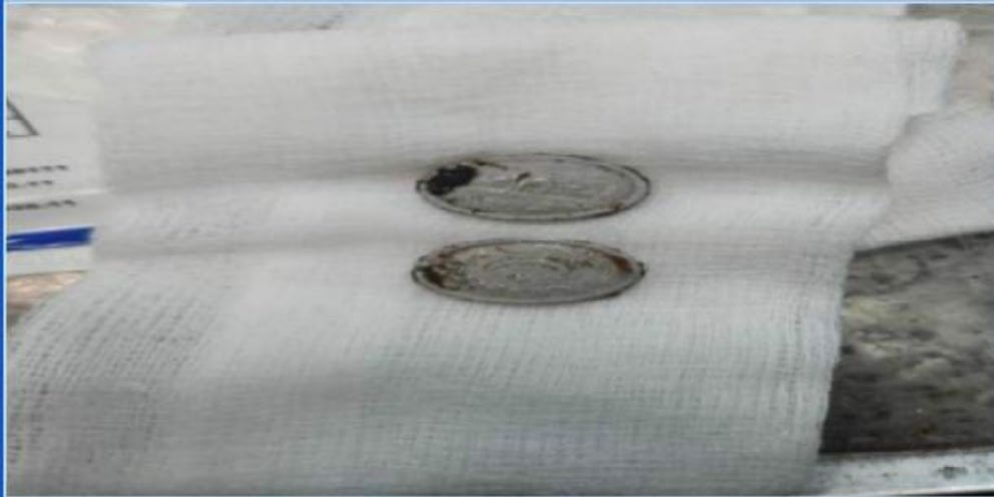
Coins retrieved per rectum.

Conservative management was initiated, including manual evacuation under sedation. Over 4 kg of impacted stool, along with ingested foreign bodies (coins and seeds), was removed (Figure 3). Intravenous hydration and electrolyte correction were administered. The patient showed gradual improvement, with resolution of abdominal distension and resumption of bowel movements. There was no need for therapeutic intervention.

### 2.4 Outcomes

Due to conservative manual excavation, the abdominal distension was resolved, prolonged constipation was relieved, and normal bowel movements were restored. There was no need for follow‐up diagnostic evaluations. The patient showed gradual improvement.

## 3 Discussion

Foreign body ingestion is a well‐documented clinical concern, especially in deaf and mute adults presenting unique diagnostic and management challenges. This case highlights unique challenges in managing gastrointestinal obstruction in a deaf and mute patient with communication barriers, which results in an obscure clinical presentation as well as delayed medical intervention. This is consistent with results from a qualitative study that examined the experiences of deaf patients in emergency departments (EDs), which showed that requesting communication access can be difficult and time‐consuming, frequently leading to inadequate therapy [7].

When it comes to the impact of ingesting foreign bodies, the majority of them pass through the GIT tract without any major issues, but many of them may cause complications. 80% of the consumed foreign things that are less than a week old leave the body without the need for surgery [8]. Less than 1% will need a medical operation, according to Louie and Bradin's gauge, while 10%–20% will need nonsurgical intervention [9]. Medical professionals working in emergency rooms face a significant problem when it comes to choosing the treatment option for deaf and mute individuals with ingested foreign bodies. Additionally, a cross‐sectional study of emergency physicians found that 88% of doctors had not received training on the topic, and 74.1% of departments lacked strategies for managing deaf patients [10].

As far as the complications are concerned, the ingestion of foreign bodies can lead to various complications, including gastrointestinal bleeding, obstruction, and perforation, which can be life‐threatening [11]. These complications are often the result of the delayed diagnosis, and such a probability increases when it comes to dealing with a deaf and mute patient with an ingested foreign body [12]. These complications that necessitate surgical intervention occur in almost 1%–5% of cases [3]. Fortunately, our patient was not presented with any complications, but his attendants gave a history of prolonged constipation, as his bowel movements were affected due to foreign body ingestion. He showed no signs of peritonitis, and his vitals were normal; all other organs and systems were working properly.

The ingestion of foreign bodies in adults is often associated with psychiatric conditions, alcohol intoxication, or cognitive impairments [4]. It is also related to pica eating, which often occurs due to iron deficiency or a lack of nutrients, leading to a craving for nonedible. Notwithstanding the usual risk profiles, the lack of such characteristics in our instance further demonstrated how crucial it is to take foreign body ingestion into account when making a differential diagnosis. However, the patient was evaluated successfully, and it was explored that there is no such history of mental or nutritional disorder, and the patient had ingested coins and seeds.

Since both are blunt or round objects, they did not cause much damage to the tract while passing through the gut. Coins are the most frequently ingested foreign body in the western pediatric population, but are also seen in adults with psychiatric disorders or prisoners. Esophageal impaction (73% of pediatric cases) requires urgent removal. Coins larger than 20 mm or that fail to pass the stomach within 3 days should also be removed. Retrieval forceps allow a quick and easy extraction [9]. With reference to the medical literature, the average length of stay for patients with foreign‐body ingestions ranges from 2 to 4 days [13].

As far as the treatment is concerned, the treatment for removal of foreign bodies depends on multiple factors such as the size of the object, the degree of sharpness of the object, the location of the object, and the surgeon's skills. Usually, after ingestion, foreign bodies are often impacted at the upper/lower esophageal sphincter, ileocecal valve, cecum, sigmoid, and anus, and often cause perforation at the ileocecal and rectosigmoid regions due to the narrowing of the bowel lumen and angulation of the digestive tract in these areas [14]. Currently, there is still no consensus on the treatment protocol for patients who have swallowed foreign bodies because the majority of cases allow the foreign bodies to pass through the digestive tract and be excreted through the rectum spontaneously. Hence, the recommendations for the initial management of foreign body ingestion are conservative treatment, close observation, and continuous imaging monitoring for about 3 days [15]. In cases where conservative treatment fails, efforts should be made to retrieve the foreign bodies via endoscopy or early surgical intervention to avoid the risk of perforation and subsequent complications [16].

We treated our patient with conservative treatment, and he showed gradual improvement in the constipation as well, following the foreign body removal, and no complications. In our case, effective communication was also the cornerstone of management. According to studies, deaf patients frequently require more than mere lipreading or written communication, which might result in miscommunication and inadequate therapy [17]. To close this gap, it has been suggested to engage qualified sign language interpreters, either in person or by video remote interpreting (VRI). Nonetheless, issues including a shortage of interpreters and inadequate staff training continue to exist [18].

This instance emphasizes how urgently healthcare systems must establish standard procedures for treating individuals who are deaf or mute. The treatment and results of patients can be improved by teaching healthcare professionals basic sign language, making sure that interpreter services are available, and creating explicit policies. It is also critical to create an inclusive atmosphere that acknowledges and meets the special requirements of patients who have communication problems.

## 4 Conclusion

Multidisciplinary care, early recognition of nonverbal cues, and tailored conservative strategies are critical in managing complex constipation due to foreign body ingestion in vulnerable and visually and auditorily impaired populations.

## Author Contributions

Muhammad Hassaan Javaid: case selection and writing the first draft. Mudassir Khalid: references, reviewing, and editing. Mohammad Yassin Al Aboud: writing, reviewing, editing, and submitting the manuscript. The authors have read and approved the final manuscript.

## Author Contributions

**Muhammad Hassaan Javaid:** conceptualization, investigation, methodology, project administration, writing – original draft. **Muddassir Khalid:** resources, writing – review and editing. **Mohammad Yassin Al Aboud:** conceptualization, investigation, writing – review and editing.

## Consent

Written informed consent was obtained from the patient's guardian for publication.

## Conflicts of Interest

The authors declare no conflicts of interest.

## Data Availability

All data and information related to this manuscript are available upon reasonable request.
